# What is the global perspective on advanced practice physiotherapy: A qualitative study across five countries

**DOI:** 10.1371/journal.pone.0320842

**Published:** 2025-04-28

**Authors:** Andrews K. Tawiah, Marguerite Wieler, Jordan Miller, Alison Rushton, Linda Woodhouse

**Affiliations:** 1 School of Physical Therapy, Faculty of Health Sciences, Western University, London, Ontario, Canada; 2 Department of Physical Therapy, Faculty of Rehabilitation Medicine, University of Alberta, Edmonton, Alberta, Canada; 3 School of Rehabilitation Therapy, Queen’s University, Kingston, Ontario, Canada; 4 Physical Therapy Department, School of Pharmacy and Health Professions, Creighton University, Omaha, Nebraska, United States of America; National Institute of Health and Medical Research: INSERM, FRANCE

## Abstract

**Background:**

Despite the growth of advanced practice physiotherapy (APP) and the evidence of its effectiveness, challenges persist in implementing and sustaining this model of care. The main challenge is the lack of a universally accepted definition. The lack of a clear understanding of what APP is, who can become advanced practice physiotherapists, and the required educational qualifications leads to widespread confusion among patients, employers, and healthcare providers.

**Aim:**

Our study aims to explore the global perspectives of advanced practice physiotherapy.

**Objectives:**

1.To understand how APP is defined and develop a common definition across countries.

2.To understand the difference between APP and clinical specialists.

3.To understand the clinical practice challenges of working as an advanced practice physiotherapist.

**Methods:**

A qualitative descriptive study was carried out through four online focus groups with participants from Australia, Canada, Ireland, New Zealand, and the United Kingdom. Participants were selected using purposive sampling, and the focus groups were recorded, transcribed and analyzed. An initial coding framework was developed by two coders, with subsequent coding performed by one of the coders. Data were analyzed using reflective thematic analysis and reported following the Standards for Reporting Qualitative Research (SRQR).

**Results:**

Sixteen participants were recruited, twelve were advanced practice physiotherapists, and four were leaders or researchers. Three major themes were developed: (1) Advanced practice physiotherapists have a higher clinical expertise and a high level of responsibility, (2) Advanced practice physiotherapists are distinct from specialists based on the competencies, scope, and regulation, (3) Professional and operational challenges are associated with APP. Ten (10) sub-themes associated with the major themes were developed and presented in a thematic map.

**Conclusion:**

This study provided a common definition of APP, distinguished advanced practitioners from clinical specialists, and discussed challenges to implementation. The study explores the complexities of APP and specialization, emphasizing the obstacles faced by practitioners. The proposed definition can be tailored to meet the specific requirements of local and regional healthcare needs. Addressing the challenges to implementation could support the sustainability of the APP model of care.

## Introduction

Many healthcare systems face significant challenges, including a lack of access to primary care, escalating wait times for patients visiting emergency departments, long wait times for surgical procedures and a shortage of healthcare professionals due to burnout and stress [1,2]. Advanced Practice Physiotherapy (APP) is seen as a model of care with the potential to address some of these most pressing challenges and future healthcare needs for patients, especially those with musculoskeletal (MSK) disorders [3–5]. APP has been embedded in healthcare systems for the past few decades and continues to expand [6,7]. Widespread adoption of team-based models of care that maximize the scope of practice of all healthcare practitioners, including physiotherapists, have positive impact at the patient, community and healthcare system levels [6–9].

### Evolution of APP

**Fig 1:**
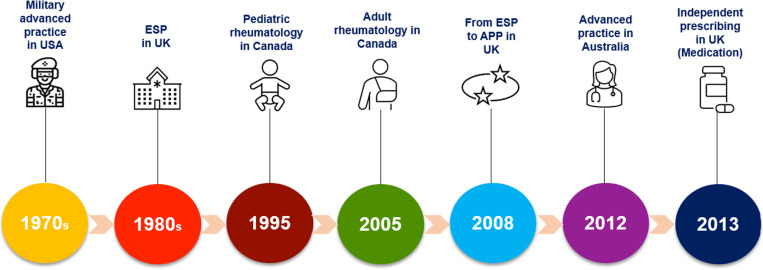
Evolution of Advanced Practice Physiotherapy. Legend: ESP = Extended Scope Practice.

APP originated in the 1970s in the United States military when physiotherapists expanded their roles to manage high volumes of injured soldiers. This development mirrored earlier advancements in the nursing profession [10]. In the 1980s, the United Kingdom adopted APP to handle increasing non-surgical caseloads and patient complexity [11,12]. Initially referred to as “Extended Scope Physiotherapists”, these practitioners took on additional responsibilities such as administering therapeutic injections, ordering diagnostic tests, and even prescribing medications by 2013 [11,12]. Canada introduced extended scope practice in 1995 at the Hospital for Sick Children in Toronto, focusing on pediatric rheumatology [13,14]. The Advanced Clinical Practitioner in Arthritis Care (ACPAC) program, launched in 2005, further developed APP roles to improve arthritis management and reduce wait times [15,16]. Australia implemented Advanced Musculoskeletal Physiotherapy (AMP) services in 2012, primarily to address the economic impact of musculoskeletal conditions [17]. This bottom-up approach to APP development has been driven by healthcare organizations seeking to tackle rising costs and improve access to care. Fig 1 is an illustration of the evolution of APP.

Recent systematic review of systematic reviews consistently demonstrates that advanced practice physiotherapists are highly effective in various healthcare settings, especially for patients with MSK disorders [18,19]. When compared to orthopedic surgeons, advanced practice physiotherapists show comparable accuracy in diagnosis, appropriate triaging abilities, and the capacity to improve treatment outcomes for patients with musculoskeletal disorders [18]. Advanced practitioners also significantly enhance access to care, often managing patients independently and reducing wait times for surgical opinions [18–20]. Additionally, advanced practice physiotherapists tend to utilize resources more efficiently, prescribing fewer medications and ordering fewer investigations compared to standard care [18]. These findings are consistent across different countries and healthcare systems, suggesting that APP services can be a valuable addition to orthopaedic care pathways, potentially reducing healthcare costs and improving patient flow [3–5,19,20].

Despite the expansion of APP and evolving evidence, there are still challenges with the implementation and sustainability of APP models of care and the role of APP in clinical practice. One of the most pressing challenges is the lack of a universally accepted definition of APP. The questions of; what APP is, who can be trained to be an advanced practitioner, how is APP different from physiotherapy specialization, and what are the educational requirements to become an advanced practitioner are not adequately addressed in the literature. The consequent lack of understanding of APP and clarity of who is an advanced practitioner is impacting the rollout of the model within and across countries. Although the World Physiotherapy policy document on APP was timely in 2019, it does not provide a definition of APP nor does it capture all aspects of APP, for example, educational pathways for training to become an APP [21]. Earlier studies (2009–2012) had provided a description of what APP is, and what advanced practice physiotherapists do; however, descriptions are already outdated considering the rapidly evolving landscape of APP [11,22,23].

Other challenges include the lack of understanding of the role of an APP which results in lack of recognition from the healthcare system, the inability of advanced practitioners to perform to their full scope of practice including ordering diagnostic investigations, and the lack of support from hospital management [24]. Previous studies investigating challenges to implementation of APP have not explored the in depth underlying reasons for these challenges. An earlier global survey among World Physiotherapy member organizations identified barriers to the implementation of APP, such as jurisdictional disputes with other healthcare professionals and a lack of legislation to support the implementation of APP [24]. A qualitative exploration study to discern the reasons behind these challenges is warranted.

Another major challenge with APP is the lack of clarity on the difference between APP and clinical specialization. A report by National Health Service, Wales suggested that differences in depth and breadth of knowledge are a distinction between advanced practice and specialization [25]. Importantly, the voice of advanced practice physiotherapists in countries with well-established models of APP has not been captured when defining APP, differentiating APP from clinical specialists and exploring the challenges with APP.

### Aim and objectives

The aim of this study was to examine the global perspectives of APP through qualitative inquiry. The specific objectives were:

1)  To explore how APP is defined from the perspective of practitioners across different countries, leading to the development of a common definition.
2)  To examine how advanced practice physiotherapists differentiate their role from clinical specialists.
3)  To identify the clinical practice challenges of working as an advanced practice physiotherapist from the perspective of the practitioners.

## Method

### Study design

This study followed a qualitative descriptive methodology with data analyzed according to thematic analysis by Braun and Clarke [26,27]. Qualitative descriptive methodology was chosen to provide a rich, detailed description and understanding of the perspectives of advanced practice physiotherapists [26]. Data from this study were collected as part of a pervious study to determine the core competency and capabilities of APP [28]. Tawiah et al. [28] identified that advanced practitioners need to develop competencies as expert clinicians, effective communicators, leaders and health advocates, scholars, and professionals. Data were collected through four online focus groups involving participants from five countries. Focus groups were conducted through Zoom with a mix of participants from Australia, Canada, Ireland, New Zealand, and the United Kingdom. Focus groups was an appropriate method for this study because it allowed practitioners from across several countries to come to the same table to discuss important issues related to their practice. This diversity of participants ensured a rich discussion and sharing of ideas and opinions.

Ethical approval for this study was obtained from the Research Ethics Board at the University of Alberta (ID: Pro00099692). All participants provided written consent, and interviews were conducted in August 2020. The study followed the Reporting Qualitative Research (SRQR) and the Consolidated Criteria for Reporting Qualitative Studies (COREQ) 32-item checklist to report and write up findings from the focus groups [29,30].

### Recruitment and participants

Purposive sampling techniques that align with qualitative descriptive methodologies were used to recruit participants from Australia, Canada, Ireland, New Zealand, and the United Kingdom. These countries were selected because they have well-established APP models of care ensuring the advanced practice physiotherapists would have the capacity to respond to the questions posed during the focus groups. The participants were individuals who were either actively practicing as advanced practice physiotherapists, had prior experience in this role, conducted research investigating APP or played a role in the advancement and growth of APP [28]. Eighteen practitioners consented to participate in the focus groups. Two advanced practitioners withdrew for personal reasons before data collection started, leaving a total of sixteen (16) participants in the focus groups. An email list of known advanced practice physiotherapists within the targeted counties were contacted. We screened them against our criteria for inclusion. After the fourth focus group, new themes emerged with a repetition of already developed themes. Data saturation was attained, and no new focus groups were conducted.

### Data collection

The research team utilized the Zoom™ video conferencing platform to conduct and record focus groups. Focus groups one to three had 4–6 participants each, while focus group four had 2 participants due to late cancellation. However, this did not affect the quality of the discussions during the fourth focus group. The principal researcher (AT) was the moderator during the focus groups, no other observer was present. Zoom automatic transcription was employed, followed by meticulous reviews of the transcripts for correctness and accuracy The transcripts were sent to the participants for review to confirm their accuracy.

To prepare for the focus group, a week before the scheduled date, the participants were sent an email package containing a letter of information, an informed consent form and a demographic questionnaire. Participants completed and emailed the consent forms to AT in advance of the focus group. Each focus group lasted about 60 minutes. A discussion guide was developed by AT and LW based on previously published studies on the definition, competencies and barriers to APP. (S1 Appendix) After each focus group, initial data analysis and preliminary coding were conducted.

### Data analysis

The data obtained from the focus groups, including raw video footage, automated transcripts, and edited transcripts, were efficiently transferred and organized using NVivo 12 (software developed by QRS International). To perform the thematic analysis, we followed the guidelines of Braun and Clark [27]. 1) Familiarization with the data 2) Generating initial codes 3) Searching for themes 4) Reviewing themes 5) Defining and naming themes 6) Producing a report. Participant’s quotes are presented with a Quotation ID. Example: F1-03-Clinician for Focus group 1 participant 3, a clinician. The findings from the focus group and the policy statement from World Physiotherapy were integrated to develop the definition of APP [21].

### Rigour and respondent validation

The lead researcher has previous experience in facilitating focus groups and conducting interviews with patients, healthcare professionals, and researchers. To ensure accuracy of the coding process, after each focus group, AT developed the initial codes from that focus group. These codes were shared with LW and MW with a sample of the transcripts to ensure that the codes were verified and accurate. Differences in codes were discussed among the research team. Once a coding framework was agreed on, AT completed coding all the remaining transcripts, this process enhanced the data analysis process. The codes and themes generated from the focus groups were shared with the participants for further clarification and validation. Respondent validation further enhanced the accuracy and trustworthiness of the data. Finally, AT kept a consistent audit trail of all coding and thematic analysis processes and met with LW and MW periodically to discuss and reflect on the coding process.

## Results

### Demographic characteristics of participants

The study involved sixteen participants from five countries, divided into four focus groups. Twelve were advanced practice physiotherapists, while five were either leaders or researchers in APP. (Table 1) About 81% had over 15 years of clinical experience, with 19% having 10–15 years and one with 5–10 years. Educationally, about 94% held graduate degrees: 8 had doctorate (PhD), seven had master’s degrees and one post-graduate diploma. (Table 1) This diverse and experienced group provided a broad perspective on APP.

**Table 1:** Participant’s demographics (n=16).

Item	Description	N (%)
APP	Yes	12 (75)
	No	4 (15)
		
Country	Canada	4 (25)
	Australia	4 (25)
	Ireland	4 (25)
	New Zealand	2 (12.5)
	United Kingdom	2 (12.5)
		
Years of experience	10-15 years	3 (18.8)
	15+ years	13 (81.2)
		
Role	Clinician	10 (62.5)
	Management	2 (12.5)
	Researcher	3 (18.8)
	Educator	1 (6.2)
		
Level of Education	Bachelors	1 (6.2)
	Masters	7 (43.8)
	Doctorate (PhD)	8 (50)

### Themes

**Fig 2:**
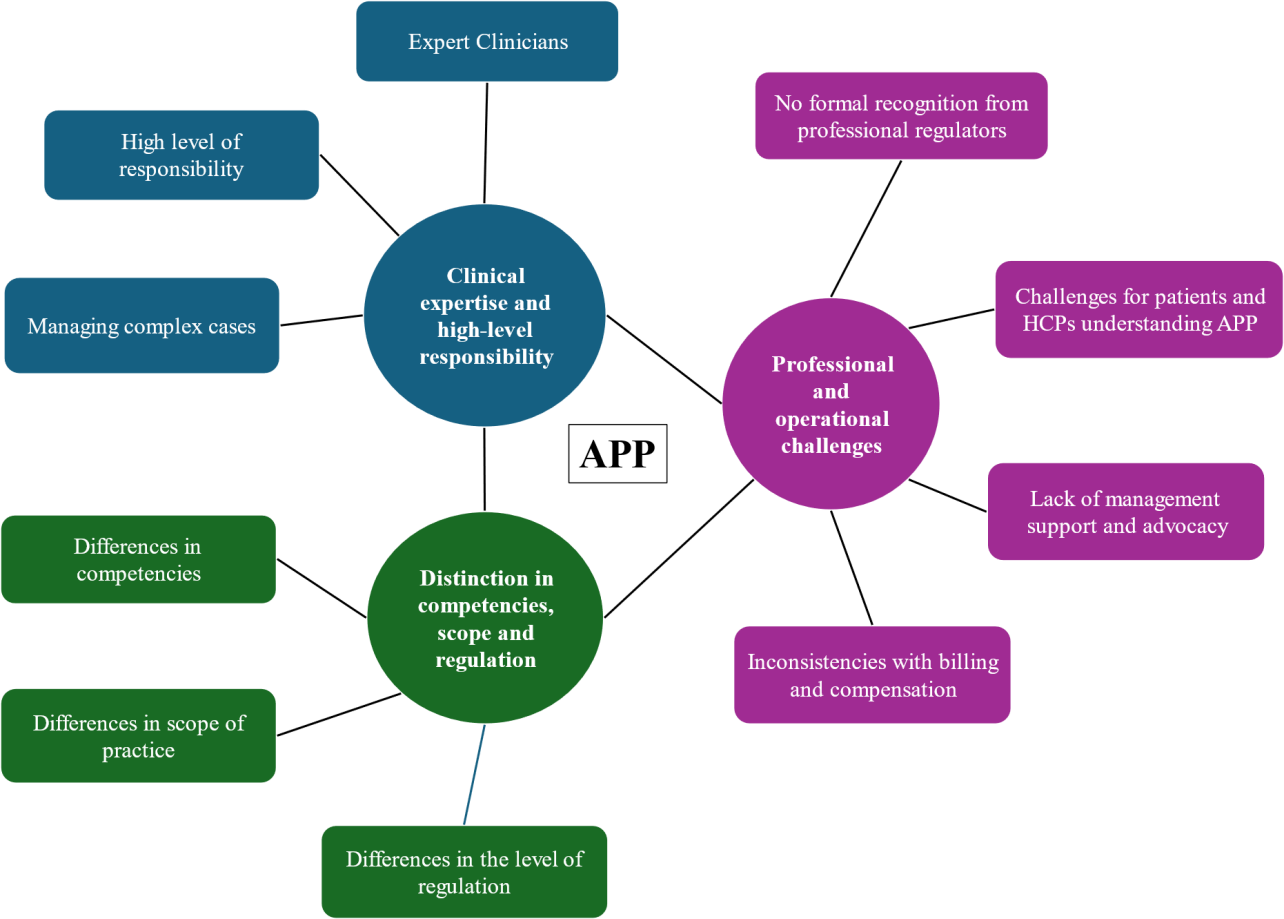
Thematic Mind Map.

Fig 2 describes the derived thematic mind map from the data analysis. The relationship between the three themes (circles) and 10 sub-themes (rectangles) developed from the data is presented. The three themes are: clinical expertise and a high-level responsibility, distinction in competencies, scope and regulation, and professional and operational challenges with APP.

#### Clinical expertise and a high-level responsibility theme.

Advanced practice physiotherapists are characterized by their extensive clinical expertise and their capacity to manage complex cases. This theme highlights how advanced practice physiotherapists operate at a high level of clinical reasoning and decision-making, often taking on roles that require advanced skills and autonomous practice in various healthcare settings. Three sub-themes emerged: **expert clinicians, a higher level of responsibility, and managing complex cases.**

**Expert clinicians sub-theme:** participants described advanced practice physiotherapists as expert clinicians, emphasizing their exceptional clinical expertise and leadership within their specific fields. They highlighted that these professionals are not only adept at managing incredibly complex cases but also serve as key consultants and leaders among their peers. Advanced practice physiotherapists were recognized for their ability to handle uncertainty and navigate the challenges of their cases, showcasing their advanced diagnostic skills and comprehensive understanding of patient care. As expert clinicians, advanced practice physiotherapists embody a high level of practice and responsibility, distinguishing themselves through their ability to provide specialized, nuanced care in complex scenarios. Example of supporting quotes are presented in Table 2.

**Higher level of responsibility sub-theme:** participants described advanced practice physiotherapists as bearing a significantly higher level of responsibility in their practice. They described advanced practice physiotherapists as being entrusted with making critical clinical decisions, including diagnostic and overall management choices, as well as handling onward referrals and discharge planning. Participants highlighted that advanced practice physiotherapists often see patients on behalf of physicians and play a crucial role in shaping management plans. This elevated responsibility also encompasses the authority to refer patients for further diagnostic imaging and specialized care. Additionally, participants noted that in regions like Ireland, the role of advanced practice physiotherapists is evolving, with these professionals primarily operating in secondary care settings such as orthopedics and rheumatology triage roles. Despite this, the definition of advanced practice physiotherapists as holding a higher level of practice, similar to the UK, underscores their integral role in the healthcare system. Example supporting quotes are presented in Table 2.

**Table 2:** Examples of supporting quotes for clinical expertise and a high-level responsibility theme.

Theme	Sub-themes	Supporting Quotes
**Clinical expertise and high-level responsibility**	**Expert clinicians**	I think I would start by saying you’re an expert clinician in whatever particular field you’re working in. So that would probably be the first and foremost thing. And you’re probably a leader right for your peers and sort of a consultant at the same time, I think. Advanced practice physiotherapists also become a good diagnostician. [F3-03-Clinician]
		I think it’s about being an expert clinician, somebody who can deal with the complexity of cases. The cases often are incredibly complex and you’re dealing with uncertainty within those cases. [F2-09-Clinician]
	**Higher Level of Responsibility**	I suppose in our role, you have a higher level of responsibility. It’s probably similar to what was said, they’re seeing patients on behalf of consultants and involved in decision making on their management plan and referral onwards. You have the responsibility or the authority to refer onwards, discharge and make the diagnostic decisions and clinical decisions on their overall management. [F1-02-Clinician]
		The Irish Society of Physiotherapists has taken on the definition as that it’s a level of practice as well, similar to the UK as opposed to a defined role. But at the moment in Ireland, those who would take on the title of advanced practice physiotherapists are only working in secondary care. So, in triage roles in orthopedics rheumatology, but that it’s also kind of a role. And that’s it’s still in development, it’s relatively new compared to the likes of the UK. [F2-05-Researcher]
	**Managing complex cases**	Advanced Practice physiotherapists combine advanced physiotherapy knowledge and skills with advanced clinical reasoning to provide person and “Whanau” [Māori term for family] centered healthcare. The advanced practitioners provide advanced assessment and management interventions such as triage for specialist clinics and second opinions for complex cases. They work across healthcare settings, influence health service delivery and the wider profession, and are involved in mentoring, supervision, teaching or research. [F2-01-Management]
		My role specifically is to triage referrals that come in from family physicians, I determined sort of the level of urgency for a consultation. Either with an advanced practice physiotherapist or whether or not they need to go directly on to an orthopedic surgeon based on the referral information. For patients that I assess clinically, I do it more on sort of a consultant-type basis. I will help to define the diagnosis by examining them, order appropriate imaging or blood work if required, and act on all those particular tests that I order as well. [F3-05-Clinician]

**Managing complex cases sub-theme:** participants described advanced practice physiotherapists as crucial in managing complex cases that often involve multimorbidity and multifaceted treatment needs. They highlighted that advanced practice physiotherapists play a key role in triaging referrals, determining the urgency of each case, and deciding whether to provide direct care or refer patients to other specialists, such as orthopedic surgeons. The participants noted that advanced practice physiotherapists use their advanced knowledge and clinical reasoning to conduct comprehensive assessments, including ordering and acting on diagnostic tests and providing second opinions for complex cases. Their expertise and advanced skills are essential for addressing the complexities of these cases, ensuring that patients receive timely and effective care tailored to their specific needs. Example supporting quotes are presented in Table 2.

#### Distinction in competencies, scope and regulation theme.

There are distinctions between advanced practice physiotherapists and specialist physiotherapists. This theme delves into the differences in competencies, scope of practice, and levels of regulation, illustrating how advanced practice physiotherapists and physiotherapist specialists fulfill unique but different roles within the physiotherapy profession. Three sub-themes emerged to differentiate between an advanced practice physiotherapist and a physiotherapy specialist: **differences in competencies**, **differences in the scope of practice** and **different levels of regulation.**

**Differences in competencies sub-theme:** participants described that a key distinction between advanced practice physiotherapists and specialist physiotherapists lies in their competencies. While specialists are recognized for their strong clinical expertise in specific areas within physiotherapy practice, advanced practice physiotherapists are expected to exhibit additional competencies beyond clinical skills in more interprofessional roles. Participants highlighted that advanced practice physiotherapists engage in leadership, advocacy, and triage roles, which are not typically emphasized in specialist roles. This includes responsibilities such as facilitating care, directing patients to appropriate services, and managing complex cases. Specialists are generally seen as expert clinicians focusing on direct patient care, whereas advanced practice physiotherapists are expected to integrate a broader skill set, including leadership and research, alongside their clinical expertise. Example supporting quotes are presented in Table 3.

**Table 3:** Supporting quotes for distinction in competencies, scope and regulation theme.

Theme	Sub-themes	Quotes
**Distinction in competencies, scope and regulation**	**Differences in competencies**	A specialist, if we go back to the FCP [First Contact Practitioner], a specialist in this case they have fulfilled that clinical pillar, but they haven’t necessarily become a master of research and a master of leadership to a point if you dissect in those boxes [F3-05-Researcher].
		What I’ve kind of come to grips with is that the specialists and I would think of it more in terms of stock condition and that expertise and the clinical skills. Whereas the advanced practice has, I suppose when you have these additional skills like leadership and advocacy [F2-12-Clinician].
		I think if I were to differentiate the competencies, I would tend to think that the specialists are probably treating clinicians, and advanced practitioners are often working in triage roles [F1-16-Educator].
	**Differences in the scope of practice**	For advanced practitioners you’re working at a high level, but outside the traditional scope of practice than the clinical specialist. The clinical specialist title are those physiotherapists who were working in maybe outpatient departments, who may not have been injecting or requesting imaging or requesting blood tests. That was the distinction [F1-07-Clinician].
		With the Advanced Practice role, you’re taking on the role of another profession, to some extent, like say the assessment that would have been done elsewhere. Whereas the specialist, traditionally, you’re working purely on physio treatments [F4-11-Researcher].
		In Ireland we had a clinical specialist role, and they were considered like specialists in rehab. When I came to my posting, we were the first considered extended scope. So, we use that old title of the ESP because we were working in triage roles with orthopedic surgeons are rheumatologists. The specialist term was almost reserved for at that stage working within the established pillars of physiotherapy [F3-08-Researcher].
	**Differences in the level of regulation**	The specialist for us with our provincial College is a protective term. So, you can’t just call yourself a specialist unless you’ve gone through some rigorous training and certification. Advanced practice, though, is different, I think you’re certified by the organization in which you work [F3-14-Management].
		You can’t use specialists unless you’ve been recognized by your association or college. Advanced Practice is a title, I guess, a looser term. Yeah. A lot of people haven’t been really trained and they call themselves advanced practice, so I think that’s what we need to do a better job is standardizing the certification piece [F2-12-Clinician].
		I would say that from a pathway perspective, like, how you are going to get those titles, you become a specialist by going through your professional pathways. Here in Australia, you go through APA. I’m sure Canada’s got one for CPA, and you go through a training program. It’s all very regimented, you have to check off certain boxes. You have to be assessed and examined, and then you can get the title of specialist [F4-11-Clinician].
		So, I think specialists have been bestowed by their professional association, and I think advanced practice is bestowed by the health care facility [F1-07-Clinician].

**Differences in the scope of practice sub-theme:** a key theme that emerged from the discussion was the difference in the scope of practice between advanced practice physiotherapists and specialist physiotherapists. Participants described advanced practice physiotherapists as having a significantly broader scope of practice compared to specialists. According to the participants, clinical specialists may not perform certain APP duties and responsibilities such as injecting, requesting imaging or requesting blood tests. This highlights the transitions of advanced practice physiotherapists working under medical directives to more independent practitioners. The distinction is further highlighted by the roles that advanced practice physiotherapists play in triage, assessment, and providing onward referrals, while specialists work on more specialized assessment and treatment within their area of specialization. Example supporting quotes are presented in Table 3.

**Differences in the level of regulation sub-theme:** participants described significant differences in the level of regulation between advanced practice physiotherapists and specialist physiotherapists. In several countries specialist physiotherapists are subject to regulatory standards and are officially recognized by national or provincial regulatory bodies. Achieving the title of specialist requires rigorous training (usually from within the profession), certification, and adherence to specific professional pathways established by associations. This title of specialist may be protected and formally regulated, ensuring that specialists meet high standards of practice. In contrast, the title of advanced practice physiotherapist may be less standardized and varies more widely across different healthcare facilities and countries. APP roles are often bestowed by the healthcare organization rather than by a formal regulatory body, leading to variability in the qualifications and education of those who hold an APP title. As a result, the term “advanced practice” can sometimes be applied more loosely, with less consistent oversight compared to the specialist designation. This lack of uniform regulation highlights a need for more standardized certification processes to ensure consistency and recognition across different regions and institutions. One participant expressed the view that the roles of advanced practice physiotherapists and specialist physiotherapists should not be considered mutually exclusive. According to this perspective, a physiotherapist can embody both roles simultaneously, integrating advanced practice skills with specialized expertise. The participant argued against creating a rigid distinction between the two roles, suggesting that combining advanced practice with specialization allows for a more comprehensive approach to patient care. This view supports the idea that a physiotherapist can achieve APP recognition while also excelling in a particular specialty, thus enriching their overall professional capabilities and contributions. Example supporting quotes are presented in Table 3.

#### Professional and operational challenges with APP theme.

Advanced practice physiotherapists face numerous challenges that hinder the full realization of their roles. Lack of recognition, regulation, difficulty understanding the role and difficulties with remuneration are examples of professional and operational challenges affecting APP. Four sub-themes emerged related to the challenges that advanced practice physiotherapists face in their practice: **no formal recognition from professional regulators, challenge for patients and healthcare professionals in understanding advanced practice, lack of management support and advocacy, and inconsistencies with billing and compensation**.

**No formal recognition from professional regulators sub-theme:** participants highlighted the lack of formal recognition for the title of “Advanced Practice Physiotherapist” by regulatory bodies in many countries. Although APP roles are accepted and valued within hospitals and healthcare settings, they are not officially recognized by professional regulatory organizations in most countries. Participants highlighted that, while the title of APP is used by physiotherapists who seek to advance patient care and push the boundaries of traditional practice, it lacks formal endorsement and standardization. This lack of formal recognition often means that advanced practice physiotherapists work under the authority of medical directives and face challenges related to how their role fits within their scope of practice. Participants from the United Kingdom described that advanced practice physiotherapists are officially recognized and that there is a structured educational pathway for these roles. Health Education England has established standards for advanced practice physiotherapists in musculoskeletal (MSK) health, and physiotherapists who meet these standards through various routes can be added to a register of advanced practitioners. However, there remains a lack of clarity regarding the specific title or designation these practitioners should use. Example supporting quotes are presented in Table 4.

**Table 4:** Quotes supporting professional and operational challenges with APP theme.

Theme	Sub-themes	Quotes
**Professional and operational challenges**	**No formal recognition from professional regulators**	Advanced practice is not currently a title in Ireland, although it is the title of choice by physiotherapists working at that level in Ireland [F2-15-Researcher].
		Yeah, so I can speak to my particular situation, I think in Ontario, Canada, the Advanced Practice physiotherapy position has not been recognized yet [F1-13-Clinician].
		I’m an advanced practitioner, which was under the New Zealand College of Physiotherapy. So, we no longer have at present, advanced practitioners. But we have [advanced practitioners] under our board which is regulated [F1-02-Clinician].
		It’s a role that’s not really recognized, we have to go through another practitioner’s provider number to provide the care. So, every decision that is made by an advanced practice physiotherapist in the emergency department goes through the provider number of the head of the emergency department [F4-10-Clinician].
	**Challenges for patients and healthcare professionals in understanding APP**	Even after five years in our emergency department, people are still confused when they come with a sprained ankle and they see a physiotherapist not a doctor. And I get called doctor a lot even though I’m wearing a shirt that says, physiotherapist and a badge that says advanced scope physiotherapist, and that can be a barrier sometimes. Other patients turn up really happy to see a physio [F2-06-Clinician].
		Certainly, the first couple of years in the emergency department were quite brutal, and it’s not until we’ve been there for a while that the value of our roles has been demonstrated to the wider emergency department clinician community that we now were accepted and some of the other craft groups have given up treating musculoskeletal when we’re around because they know they just can’t compete. We’re not competing with them, though, we’re trying to work with them to put the right patient for the right clinician, basically, and we’re not always the right clinician, sometimes, we need a team working on a patient, and sometimes the patient is not for us [F2-12-Clinician].
		I would say patients don’t really understand it. I think what they hope to get is an expert within the specialty or that field. But if you talk about advanced practice, they probably don’t really understand what that means. So, I think the terminology around what we call ourselves is problematic from a patient’s point of view [F1-07-Clinician].
		From other professions, there’ll be some professions that will understand it because they’re going down the same kind of route. Some of the allied health professionals will understand it. And possibly depending on where you’re working, then obviously those people working if you’re working in the hospital, the surgeon might understand it because that’s your title. But I think overall, there’s not really a strong understanding of what we do. I would say or what that means [F2-04-Educator].
		Here in Ontario. I don’t think we’re consistent in the term that we’re using. Our primary referral sources are general practitioners (GPs), primary care providers and they really still many do not understand why their patients are not going directly to see a surgeon, and I didn’t request a physical therapist. Why are they seeing a physio that wasn’t my request? But they don’t have a good grasp of where you need them to be. [F1-13-Clinician]
	**Lack of management support and advocacy**	And management is probably the most difficult group because we have a succession of different leaders in our physiotherapy department. And not one of them has recognized the difference between the advanced scope roles and all the other roles in the hospital and we’ve had a number of moments where they’ve tried to just include lead cover for instance, by just using another physio from the hospital who hasn’t worked in a decision-making role to come in and just cover us. And so even though we’ve been going for five years. We’ve still got a lot of work to do to educate people. [F3-14-Clinician]
		Well, certainly in the first few years we were there, we didn’t have the same supports admin wise as the medics would have gotten even though we were actually working on behalf of the medics seeing their patients. [F4-10-Clinician]
		I think it’s always said when within the profession we end up having competing interests, rather than actually everyone working towards the same thing. So yeah, I think that’s where the association definitely needs to take leadership. We don’t have the college at the moment, which is a shame. But yeah, you’d like to think the college and the association will be working together [F2-09-Management]
	**Inconsistencies with billing and models of remuneration**	We don’t have as far as I know, we don’t have a provider number for [advanced practice physiotherapists]. So, we can’t bill as such. [F2-12-Clinician]
		I think we’re fighting desperately to maintain a level of pay that you know, it is right for what we do. I think it’s really challenging. [F3-14-Clinician]
		I think a lot of advanced practice physiotherapists are fighting for proper remuneration and I think there should be some standards there as well. [F4-10-Clinician]

**Challenges for patients and healthcare professionals in understanding APP sub-theme:** participants described the significant challenge of understanding APP roles, both for patients and healthcare professionals. Participants noted that patients often confuse advanced practice physiotherapists with physicians, despite clear labelling and credentials indicating their actual role. This confusion can lead to misunderstandings and frustration, particularly when patients expect to see a physician but are instead seen by an advanced practice physiotherapist. Participants highlighted that patients may have difficulty grasping the specific role and expertise of an advanced practice physiotherapist, leading to mismatched expectations. This confusion extends to other healthcare professionals. Initially, many colleagues in clinical settings were uncertain about the role and value of advanced practice physiotherapists. Over time, however, as the role’s contributions became more evident, acceptance and understanding grew. The integration of advanced practice physiotherapists into healthcare teams often requires ongoing education and clarification to ensure that both patients and colleagues fully comprehend and appreciate their role and expertise. Example supporting quotes are presented in Table 4.

**Lack of management support and advocacy sub-theme:** participants described the lack of management support and advocacy for advanced practice physiotherapists from both within and outside the physiotherapy profession. They described significant challenges in implementing and expanding APP models of care due to inadequate recognition and support from hospital management and leadership. Despite their ongoing contributions, advanced practice physiotherapists often face obstacles such as being undervalued compared to their medical counterparts, including receiving less administrative and research support and having their roles misunderstood or inadequately covered by other physiotherapists who lack experience in decision-making roles. Participants also highlighted the need for stronger advocacy from professional associations. There was a consensus that the absence of a coordinated effort from associations has hindered progress in advancing the recognition and support for APP roles. The lack of a unified voice and leadership in advocating for advanced practice physiotherapists has contributed to difficulties in integrating their roles effectively into healthcare systems and securing the necessary support to optimize their impact. Enhanced advocacy and support from professional bodies are seen as crucial for addressing these challenges and ensuring the successful implementation and expansion of advanced practice models. Example supporting quotes are presented in Table 4.

**Inconsistencies with billing and compensation sub-theme:** participants described significant challenges in billing for their services due to the lack of a dedicated provider number for advanced practice physiotherapists, which hampers their ability to claim reimbursement for their work. This administrative gap contributes to ongoing struggles with fair compensation.

Participants also highlighted that their remuneration often does not align with the advanced level of practice and high level of responsibility they undertake. Despite their critical role and the complexity of their work, many advanced practice physiotherapists find themselves fighting to secure appropriate pay that reflects their expertise and contributions. The absence of standardized billing codes and clear remuneration models for APP creates disparities and inequities, making it difficult for these professionals to be compensated fairly for their advanced roles and responsibilities. Example supporting quotes are presented in Table 4.

#### Definition of APP.

Based on the findings from the focused groups, a definition for APP should include the following main constructs: (i) expert clinicians (ii) higher level of responsibility (iii) managing complex cases. These three main constructs, together with the World Physiotherapy policy statement of APP, show that physiotherapists in advanced practice roles have received additional post-licensure education, managed complex and challenging health needs, and have a higher level of clinical expertise [21]. The following common definition of APP is proposed:

*“APP is a broad term that refers to expert physiotherapists who employ a higher level of competencies and expertise with additional responsibilities and autonomy to manage complex and challenging health needs of individuals, families and populations within or beyond their scope of practice. Advanced practice physiotherapists demonstrate competencies as expert clinician, communicator, collaborator, leader, health advocate, scholar, and professional”.*

## Discussion

This study explores what it means to be an advanced practice physiotherapist by directly asking advanced practice physiotherapists through a series of online focus groups. The study focuses on how they define advanced practice, how they differ from specialists, and the challenges they face. The research participants are experienced advanced-practice physiotherapists who have worked across various models of care, including arthroplasty triage, emergency departments, and specialized clinics.

### Definition of advanced practice physiotherapy

The findings suggest that advanced practice physiotherapists can be defined based on three main constructs, i.e., **as expert clinicians** with a **higher level of responsibilities** and **managing complex cases**. This study found that physiotherapists working at the advanced level are expert clinicians with advanced clinical reasoning and decision-making skills, which allows them to make complex decisions in different clinical settings such as emergency departments, surgical triage, rheumatology clinics etc. Previous studies have found the clinical decision-making skills (diagnostic accuracy) of advanced practice physiotherapists to be comparable to those of medical experts, including orthopedic surgeons [3,31–33]. Our study aligns with previous research on the importance of a high level of clinical expertise for advanced practice physiotherapists [5,34].

APP is a level of practice where practitioners have higher responsibilities and autonomy with patient care. This is consistent with previous studies which have described advanced practice as a level of practice. [35,36] Additionally, the ability to autonomously assess patients and provide onward referral or list for surgery has been identified as a characteristic of APP [5,37,38]. Our study aligns with previously published studies on the complexity of cases managed by advanced practice physiotherapists [39]. Advanced practitioners in triage roles or the emergency department manage complex cases requiring urgency and high clinical judgment. Tawiah et al. [28] found that advanced practice physiotherapists need to develop competencies as expert clinicians, effective communicators, leaders and health advocates, scholars and professionals. In the World Physiotherapy policy document on advanced practice physiotherapy, the need for a significant level of experience, collaborative work, research, knowledge translation, leadership in service delivery and additional education and competencies distinct from that of entry-level physiotherapists are proposed [21].

The common definition of APP proposed in this study highlights the importance of practitioners operating at a highly proficient level and demonstrating competencies and capabilities that fall within the scope of their regulated practice. This definition is consistent with previous conceptualizations of APP and is applicable across various countries and jurisdictions. [17,40,41] Furthermore, our definition of APP conforms with previous definitions from the United Kingdom, Australia, Canada, and Ireland [17,41,42].

### Difference between advanced practice physiotherapy and specialist

Another key discovery from our research is the contrast between advanced practice physiotherapists and physiotherapy specialists. The physiotherapy community is currently discussing whether there is a difference between advanced practice and specialists. This debate often ends in further confusion about the need for a clear definition of APP. The findings suggest that advanced practice physiotherapists and clinical specialists have three levels of distinction: competencies, scope, and regulation.

**Fig 3:**
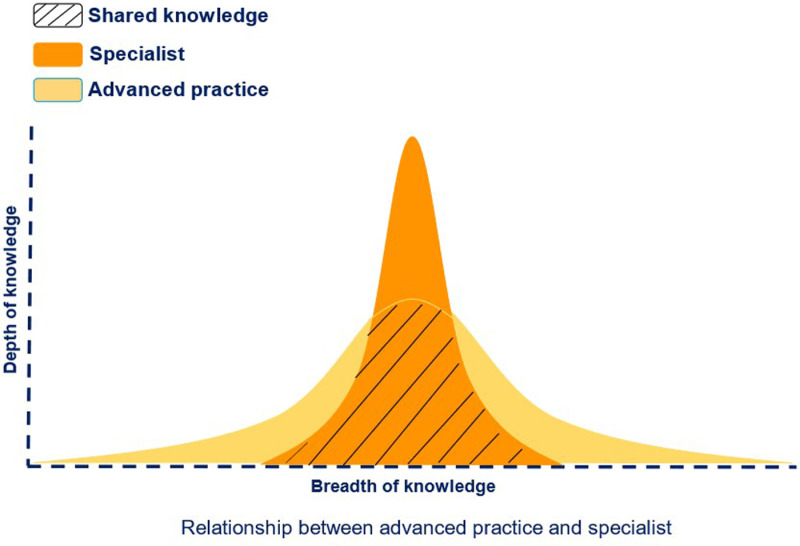
Difference between APP and Specialist (Adapted from NMAHP Framework) [ 25].

These findings suggest that advanced practice physiotherapists have additional competencies and capabilities that clinical specialists might not need to demonstrate. For example, although clinical specialists are focused primarily on clinical competencies, advanced practitioners need to demonstrate additional competencies in leadership, scholarship, communication, and advocacy for the patient, families, groups, communities, and populations [25,41]. Clinical specialists are considered to be working within the recognized scope of physiotherapy practice with an emphasis on in-depth clinical assessment and patient management, while advanced practice physiotherapists are considered to be working within a broader scope of practice, as depicted in Fig 3 [25,40]. The findings from this study are consistent with previous studies and reports that have characterized advanced practice as having a broader breadth of practice, while clinical specialists have a much narrower breadth of practice but a higher depth of practice [12,25]. These findings could be attributed to advanced practice working mostly in triage roles in orthopedics and MSK conditions and providing onward referrals when appropriate.

Clinical specialist physiotherapists have long been integrated into the physiotherapy profession with well-established pathways to becoming a specialist in several countries [43]. Clinical specialists have clearly laid out levels of regulation with the physiotherapy profession, which is often not the case for advanced practice physiotherapy across several different countries [43,44]. This is not the case for APP where the educational pathways and levels of regulation are currently not well outlined in most countries.

### Challenges to advanced practice physiotherapy

There are several different challenges encountered by advanced practice physiotherapists. Our study found that the lack of recognition from professional regulators is a challenge for practitioners. Most advanced practice physiotherapists are not recognized by the regulators for their level of skills and competency. This is in contrast to the findings from a survey by The Health and Care Professions Council in the UK on the need for regulation of advanced practice [45]. They found that practitioners were skeptical of the need for more regulation of advanced practitioners. The report found that more regulation of advanced practitioners may not correlate to better patient outcomes, and regulation may serve as another form of red tape and limit advanced practitioners [45]. An argument in favour of the need for professional regulation is to ensure the sustainability of the model of care and its acceptance by other healthcare providers. However, the merits of this argument should be weighed against the additional red tape and barriers that regulation may bring to APP.

Another important finding from this study is that inconsistency with the scope of practice for advanced practice physiotherapy is a challenge which could impact how patients and other healthcare professionals understand advanced practice physiotherapy. Because advanced practice was initially referred to as extended-scope practice, practitioners were considered to be working outside their traditional scope of practice. However, with clarification around the scope of practice across most countries, advanced practitioners are considered to be working at a higher level within their scope of practice rather than outside their scope of practice [40]. Inconsistencies with the scope of practice affect the implementation and sustainability of APP. Inconsistencies with the scope of practice and definition of advanced practice often result in the lack of support from managers within healthcare organizations to develop or expand advanced practice physiotherapy. Previous studies have also identified the lack of management support and understanding of the role as important barriers to implementing APP [24,46].

Billing and compensation for advanced practice physiotherapists were identified as a challenge to practitioners. Advanced practice physiotherapists consider the lack of provider numbers to bill patients through their services and the need for an appropriate level of compensation as challenging. Billing and compensation for advanced practice physiotherapists has not been adequately explored. A previous survey across the UK found inconsistency and pay discrepancies among advanced practitioners [36]. It is important to note that advanced practice physiotherapists advocate for appropriate compensation for their level of practice. The UK band system ensures that advanced practitioners are placed at a higher band and receive the appropriate compensation [36]. This is not the same for many other countries which do not place advanced practice physiotherapists at a higher level. The inadequate compensation for the level of practice has the potential to impact the scale and spread of APP models of care.

### Strengths and limitations

This study explored APP from five countries, which led to a common definition of APP using focus groups. A consensus-building methodology such as a Delphi or Nominal Group Technique could have been used to explore APP. These five countries were included in the study because they have well-established advanced practice roles and could provide insight for other countries to build on. The inclusion of practitioners from 5 countries strengthens the validity of the study. One limitation is that these 5 countries are highly developed and well-funded healthcare systems. The findings may not be applicable to countries with limited resources and less developed healthcare systems. Caution should be taken when generalizing the findings to these countries. Another limitation is that data for this study was collected about 4 years ago; however, we are confident that the data is still relevant to contemporary advanced practice physiotherapy based on the unique findings.

## Conclusion

The implementation of APP has the potential to address pressing healthcare concerns such as insufficient accessibility, lengthy wait times, and workforce shortages. One of the key challenges to scaling and spreading APP is the need for a common global definition and an acknowledgement of how it differs from clinical specialists. This explores how practitioners define APP, leading to the development of a common global definition for APP. The proposed definition of APP can be tailored to fit the unique needs of local and regional healthcare systems.

Addressing the challenges and barriers to the implementation of APP could lead to the scale and spread of the model of care to improve patient outcomes. This study is pivotal to shaping the future direction of APP and impacting patient care across different healthcare settings.

## Supporting Information

S1 Appendix Discussion Guide. (DOCX)
