# Supplementary material for: What is the global perspective on advanced practice physiotherapy: A qualitative study across five countries
Source: PLoS One. 2025 Apr 28;20(4):e0320842. doi: 10.1371/journal.pone.0320842 (PMC12036923; doi:10.1371/journal.pone.0320842)
Supplement: S1 Appendix — (DOCX) [file pone.0320842.s001.docx]

Supplementary Materials

**Appendix 1**: Discussion Guide

| **Discussion Guide** |
| --- |
| **Advanced Practice Physiotherapy Focus Group** |
| **Researchers involved:** |
| **Andrews Tawiah:** leading the focus group |
| **Andrews Tawiah:** Take procedural notes and suggestions for changes with the next group |
|  |
|  |
| **Start Recording** |
|  |
| **Plan:** |
| 1. Welcome participants, information and consent form, and a demographic questionnaire. |
| 1. Both consent forms and demographics questionnaires will be sent out prior to the day of the focus group. |
| 1. Description of the project and summary of what we have found so far: |
| - - - - A summary of the first draft of competencies will be sent out to the participants prior to the day of focus. |
| 1. Objectives of the focus group; |
| 1. Description of the plan of the focus group; |
| 1. Questions: |
| **Questions** |
| 1. How do you define Advanced Practice within jurisdiction? |
| - How well do you think patients, other healthcare professionals and healthcare management within your jurisdiction understand this role? |
|  |
| (Provide a description of the 6 roles) |
| 1. Within your jurisdiction, what is the difference between the roles compared to entry-to-practice? |
| - Expert Clinician |
| - Communicator |
| - Collaborator |
| - Leader and Health Advocate |
| - Scholar |
| - Professional |
|  |
| 1. Which of these 6 roles do you perceive to be the most crucial in establishing advanced practice roles compared to entry-to-practice? |
| - Can you provide some reasons to support your selection? |
|  |
| 1. (Focusing on Competencies) |
| For each role: which competencies do you consider key. |
| - - (Give an example of competencies) |
| - - PowerPoint slides of competencies on screen |
| - - Can you provide some reasons to support your selection? |
|  |
| 1. Focusing on APP and Specialist |
| How can we distinguish the differences in competencies between APP and Specialists based on your jurisdiction? |
|  |
| 1. Is there anything else that you would like to include? |
